# Leg massage during pregnancy with unrecognized deep vein thrombosis could be life threatening: a case report

**DOI:** 10.1186/s12884-020-02924-w

**Published:** 2020-04-22

**Authors:** Krongkarn Sutham, Sukumpat Na-Nan, Salilthip Paiboonsithiwong, Pakorn Chaksuwat, Theera Tongsong

**Affiliations:** 1 Department of Emergency Medicine, Chiang Mai, Thailand; 2 Department of Obstetrics and Gynecology, Faculty of Medicine, Chiang Mai University, Chiang Mai, 50200 Thailand

**Keywords:** Deep vein thrombosis, Massage, Pregnancy, Pulmonary embolism

## Abstract

**Background:**

Traditional massage seems to be safe but not entirely risk free, though serious adverse events are very rare. This report is aimed at illustrating a rare but fatal presentation of massive pulmonary embolism caused by leg massage and also to encourage both massage providers and pregnant women to be aware of undetected or subtle deep vein thrombosis, which could be a life threatening condition as a consequence of leg massage.

**Case presentation:**

A 25-year-old primigravid Thai woman underwent massage at a traditional massage shop at 25th week of gestation. Shortly after leg and foot massage, she had a sudden onset of dyspnea, followed by consciousness alteration, brief spastic-like convulsion, became unconscious and suffered a cardiac arrest. Basic life support (BLS) at the event scene as well as during transfer and advanced cardiovascular life support (ACLS) at the hospital were provided, resulting in successful resuscitation but persistent coma. Bedside echocardiography showed poor contractility of the dilated right ventricle, and pulmonary embolism was suspected. CT angiography (CTA) revealed multiple concentric intraluminal filling defects within the right and left pulmonary arteries, indicating massive pulmonary embolism. The fetus died in utero and spontaneous labor and vaginal delivery occurred.

**Conclusion:**

Leg massage in patients with deep vein thrombosis can dislodge thrombi, leading to life threatening pulmonary embolism, and should be contraindicated. Since pregnant women are at a higher risk of undetected or subtle thromboembolism, traditional massage in pregnant women should be contraindicated unless they are proven to have no such risk.

## Background

Massage is generally defined as the systematic manipulation of soft tissues of the body for alleviating pain, muscle relaxation or therapeutic purposes as alternative medicine. Presently, traditional massage is widely practiced and has become popular. The massage consists of several techniques of maneuvering, such as stroking and gliding, percussion, and kneading. Traditional massage is becoming increasingly popular as both a relaxation lifestyle and alternative medicine. However, the serious/fatal complications secondary to the methods are of less concern and rarely mentioned elsewhere. In our country (Thailand), traditional massage is a widely used massage technique and is currently accepted by the local Thai Ministry of Public Health. Nevertheless, the effectiveness and safety of this technique are not well known. Though systematic reviews on the effectiveness of massage therapy have demonstrated positive conclusions, adequate risk-benefit evaluations are not feasible, and several case reports indicate that it is not entirely risk free, though serious adverse events are probably rare [1]. Pulmonary embolism is the most serious consequence of massage, which has rarely been reported [2–4]. To the best of our knowledge, this is the first case of traditional massage causing massive pulmonary embolism in a healthy pregnant woman. Therefore, we report this case, following the CARE guidelines, mainly with the aim of describing a very rare but fatal presentation of massive pulmonary embolism secondary to leg massage. Also, we aim to encourage both professional massage providers and pregnant women to be aware of undetected or subtle deep vein thrombosis, which could be a life threatening condition as a consequence of leg massage.

## Case presentation

**Fig. 1:**
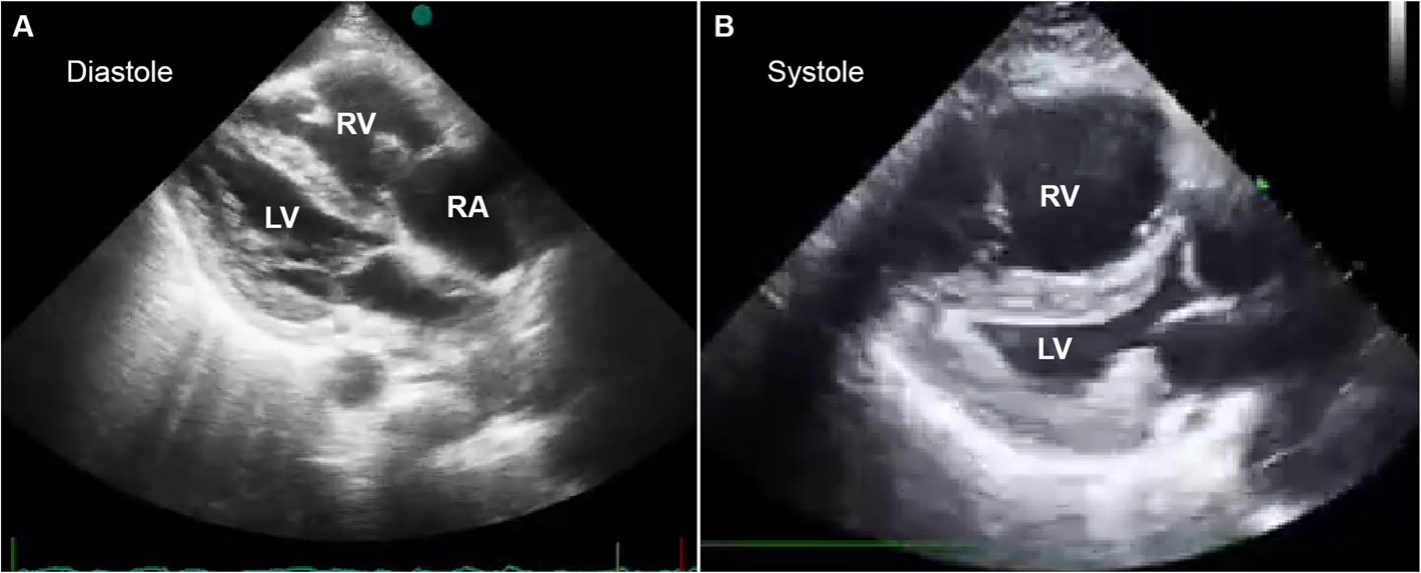
Bedside echocardiography shows poor contractility of the dilated right ventricles during diastole **a** and systole **b**

**Fig. 2:**
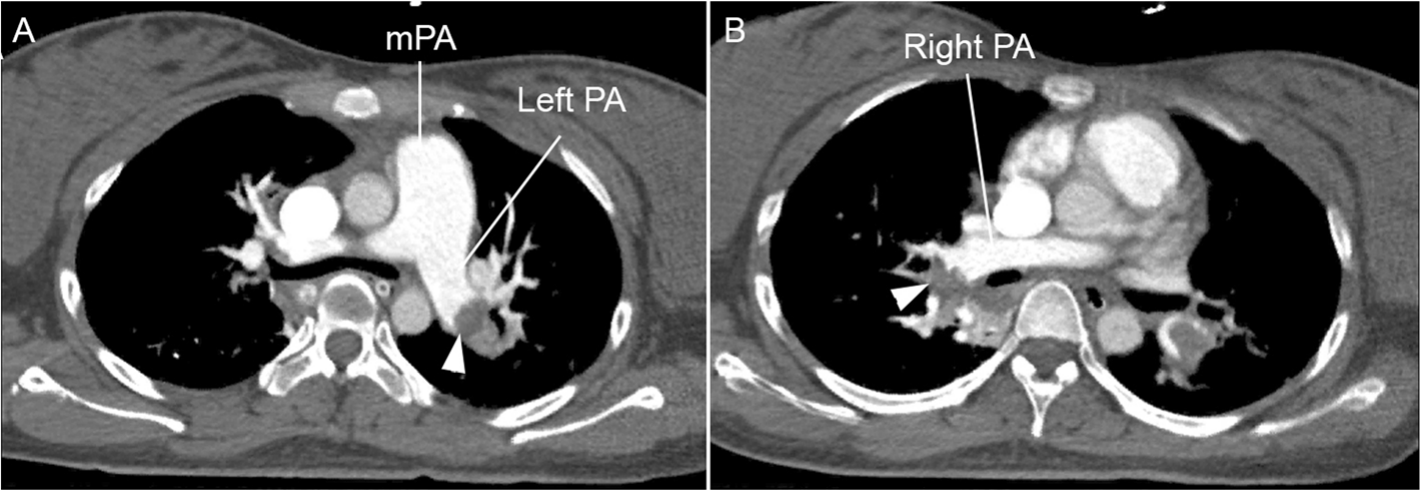
CTA of the chest (cross-section) shows large focal perfusion defects in the right **a** and right **b** pulmonary artery, representing bilateral pulmonary emboli

The patient’s background is as follows: She was a 25-year-old primigravid Thai woman. Her obstetric data was limited, since she had never attended prenatal clinic before. Most of her obstetric history was obtained from close relatives. She was a low-risk pregnancy, without any known underlying diseases and with no history of significant familial diseases. The pregnancy course before the event was uneventful, but had not been documented. She attended a traditional massage service at a commercial relaxation massage shop at a shopping center in Chiang Mai, Thailand. The traditional Thai massage is a physical form of massage involving yoga-like movement, stretching, and application of direct pressure with the assistance of the massage performer. The client wears loose clothing and lies down on a padded mat on the floor, going through several poses. The performer may use his/her hands, forearms, knees, elbows, or feet to flex the joints and apply direct or rhythmic pressure to the client’s muscles without the use of oil or lotion. Each session usually takes one or 2 hours. Approximately 5–10 min after the leg and foot massage, the woman had a sudden onset of dyspnea, followed by consciousness alteration, brief spastic-like convulsion, became unconscious and suffered a cardiac arrest. First aid and resuscitation were provided by two nurses who were incidentally at the scene. The emergency medical service (EMS) of Maharaj Nakorn Chiang Mai Hospital was emergently called and arrived at the scene of the event within 20 min, while the patient was pulseless. The EMS team performed basic life support (BLS), endotracheal intubation and continuous cardiopulmonary resuscitation (CPR) at the scene and during transfer to the hospital. About 25 min after initiation of CPR, the patient arrived at the hospital. Electrocardiogram (ECG) indicated pulseless electrical activity. Laboratory tests on admission were as follows: Hb: 11.7 g/dL; Hct: 36.0%; WBC: 12,850 cells/cu.mm; neutrophil: 50.0%; lymphocyte: 44.8%; nucleated red blood cell: 0.3/100 WBC; platelets: 159,000 cells/cu.mm. Coagulation tests gave the following results: PT: 10.50 s; INR: 0.95; PTT: 29.70 s; PTT ratio: 0.93; fibrinogen: 100 mg/dl; blood lactate: 3.14 mmol/L. Urinalysis results were within normal limits. Arterial blood gas showed severe metabolic acidosis. At the emergency room, advanced cardiovascular life support (ACLS) was immediately performed by the emergency physician team, and successful resuscitation was achieved, though the patient was still unconscious or comatose and depended on a ventilator. Bedside ultrasound after successful CPR at the emergency room showed no intra-abdominal free fluid, no pericardial/pleural effusion, no visualized aortic flap, normal left ventricular ejection fraction, hyperdynamic heart, right ventricular dilatation with poor systolic function, and interventricular septum shifted to the left, indicating right heart overload (Fig. 1, Video 1). Due to the sudden onset of dyspnea followed by cardiovascular collapse during leg massage and poor right ventricular function, pulmonary embolism was highly suspected. CTA of the chest was emergently requested and revealed multiple concentric intraluminal filling defects within the right and left pulmonary arteries and almost the entire branches that supply both lower lungs. Multiple intraluminal filling defects within segmental branches in the right middle lung and both upper lobes as well as a few broad base wedge-shaped hypoenhancing consolidation of pulmonary infarction in both upper lobes and superior segment of the right lower lung were also noted. The findings indicated massive pulmonary embolism (Fig. 2, Video 2). CT brain scan, performed to exclude neurological disorders, revealed normal results. Compression ultrasonography of both legs indicated deep vein thrombosis. Due to hypoxic encephalopathy, the CVT (Cardio-Vascular-Thoracic surgery) team decided not to perform embolectomy. However, the patient received conservative medications (Rt-PA: recombinant tissue plasminogen activator, Levophed, etc.). About 10 min after the return of spontaneous circulation, obstetric assessment was done. The ultrasound examination on admission revealed a single live fetus (fetal heart rate: ~ 130 bpm) without structural anomaly, consistent with 25 weeks of gestation, normal grade-1 placenta located at the posterior wall and normal amniotic fluid volume. Dexamethasone for fetal lung maturation was given (6 mg IM). However, 2 hours and a half later, fetal distress was observed and intrauterine resuscitation was provided. The risk and benefit of cesarean section due to fetal distress was comprehensively discussed by the care team, and the option of cesarean section was offered, but the relatives decided against operation to minimize the additional risk of the patient from cesarean section. The fetus died in utero within 8 h of fetal distress, and spontaneous labor occurred, leading to vaginal delivery without intrapartum and postpartum complication. At the time of writing this report (2 months after the event), the patient was still unconscious.

## Discussion and conclusion

Due to sudden onset of dyspnea, consciousness alteration and cardiovascular collapse during massage manipulation of the patient’s leg, which was later proven to have pulmonary embolism and deep vein thrombosis, it is reasonable to conclude that the patient had undetected preexisting deep vein thrombosis, which was mechanically dislodged by the massage and travelled massively to both lungs, leading to a life threatening condition. However, differentiation from other causes of sudden dyspnea should be considered, including heart failure, ischemic heart, pericarditis, pneumonia, pneumothorax, exacerbation of chronic lung disease, and musculoskeletal pain [5–8]. For patients suspected of pulmonary embolism, tests including ECG, chest film, brain natriuretic peptide and troponin levels, and arterial blood gases are often helpful in differential diagnosis and management [5–8]. However, a diagnosis of pulmonary embolism should be confirmed by CTA or magnetic resonance pulmonary angiogram. Patients who are highly suspected of having this condition and unstable hemodynamics must be fully resuscitated, given anticoagulation and diagnostic imaging. For cases that remain unstable despite resuscitation, bedside echocardiography and compression ultrasonography with Doppler of the leg veins should be used to make a rapid diagnosis to justify the administration of potential life-saving treatment, including thrombolytic agents [5–8].

The important clinical learning point gained from this case report is that massage of the lower extremities in cases of deep venous thrombosis is contraindicated as it can dislodge the thrombus and can cause a life-threatening pulmonary embolus. More importantly, pregnant women are in a physiologic hypercoagulable state and are at a higher risk of thromboembolism. Pregnancy can place patients with preexisting undetected or subtle deep vein thrombosis at a higher risk of pulmonary embolism. Therefore, pregnant women should be advised to avoid leg massage unless they are certain that no thromboembolism disorders exist. This case report should encourage professional massage providers to be aware of subtle preexisting deep vein thrombosis, especially in pregnant women. This case also provides an additional learning point by illustrating the role of bedside echocardiography in right ventricular evaluation in massive pulmonary embolism. Though echocardiography is generally not considered a diagnostic tool for pulmonary embolism, in unstable patients with massive pulmonary embolism, echocardiography for right ventricular evaluation can be used as a diagnostic tool. Furthermore, echocardiography is also useful in risk categorization and prognosis in pulmonary embolism. The echocardiographic features suggesting pulmonary embolism are poor contractility of the right ventricle, right ventricular dilatation, tricuspid regurgitation, paradoxical motion of the interventricular septum, pulmonary artery dilatation, elevated pulmonary pressures, empty left heart and, rarely, a right heart thrombus [4].

The timing of symptom development in relation to trigger activity was a very unique feature. The onset of cardiovascular collapse was shortly after leg massage, highly suggestive of thrombus embolization. Unlike most previous case reports of leg massage, concerning induced pulmonary embolism that occurred in previously known cases of deep vein thrombosis in non-pregnant patients [2–4], the case presented here is the first case report of leg massage leading to thrombi dislodge with the consequence of severe morbidity in a healthy pregnant woman who had undetected preexisting deep vein thrombosis, which was likely aggravated by physiologic changes during pregnancy. Unfortunately, our patient had not attended antenatal clinic before the event. Thus, she had no chance of undergoing thrombotic risk assessment (Caprini score) in early gestation. If the assessment showed high risk of thromboembolism, preventive anticoagulant might have been helpful. It is noteworthy that not only the mother but also the fetus life was threatened following pulmonary embolization. Fetal distress was detected shortly after admission. This is not unexpected since fetal distress is common after catastrophic events leading to hypoxia in the mother. Certainly, some degree of maternal hypoxia can cause a decrease in placental perfusion, resulting in fetal distress and death finally. In spite of maternal improvement after resuscitation, fetal distress may not always subside because of prior prolonged fetal asphyxia. The resuscitation improved hemodynamics in maternal vital organs but did not restore placental circulation. The decision of cesarean section due to fetal distress in this case was challenging. Several concerns were taken into considerations; for examples, the survival rate of the baby at 25 weeks of gestation in our center, which was about 25%, or much lower in cases of fetal distress, together with high morbidity, additional risk to the patient secondary to cesarean section or hemorrhage which could worsen her life-threatening condition. We followed the standards of care and medical ethics, comprehensive counseling was provided by the care team and the decision was based on the principles of autonomy and nonmalefficience.

In our country, traditional massage is widely practiced without proper guidance by local governmental agencies. Accordingly, several laypersons without adequate training practice traditional massage, and many massage shops are operated without governmental control. Similar to any other medical therapy, traditional massage may be associated with adverse effects, and it is an issue that needs to be studied further. Based on this report and literature review, strict control of our traditional massage by governmental agencies is needed, and the practice needs standardization.

In conclusion, leg massage in patients with deep vein thrombosis can dislodge thrombi, leading to life threatening pulmonary embolism; therefore, it should be contraindicated. Since pregnant women are at a higher risk of undetected or subtle thromboembolism, traditional leg massage in pregnant women should be contraindicated unless they are proven to have no such risk.

## Supplementary information

**Additional file 1: Video 1.** Bedside echocardiography shows poor contractility of the dilated right ventricles and paradoxical motion of the interventricular septum

**Additional file 2: Video 2.** CTA of the chest (cross-section) shows large focal perfusion defects in the right and right pulmonary artery, representing bilateral pulmonary emboli.

## Abbreviations

ACLS: Advanced Cardiovascular Life Support
BLS: Basic Life Support
Bpm: beat per minute
CPR: Cardiopulmonary resuscitation
CTA: CT Angiography
CVT: Cardio-Vascular-Thoracic surgery
EMS: Emergency medical services
PE: pulmonary embolism
Rt-PA: recombinant tissue plasminogen activator
